# Mechanisms of ginsenosides exert neuroprotective effects on spinal cord injury: A promising traditional Chinese medicine

**DOI:** 10.3389/fnins.2022.969056

**Published:** 2022-08-23

**Authors:** Le Qi, Jun Zhang, Jinghong Wang, Junyan An, Wu Xue, Qinyi Liu, Yan Zhang

**Affiliations:** Department of Orthopedics, The Second Hospital of Jilin University, Changchun, China

**Keywords:** ginsenosides, spinal cord injury, traditional Chinese medicine, mechanism, neuroprotective effect

## Abstract

Spinal cord injury (SCI) is a devastating disorder of the central nervous system (CNS). It is mainly caused by trauma and reduces the quality of life of the affected individual. Ginsenosides are safe and effective traditional Chinese medicines (TCMs), and their efficacy against SCI is being increasingly researched in many countries, especially in China and Korea. This systematic review evaluated the neuroprotective effects of ginsenosides in SCI and elucidated their properties.

**Methods:**

All experimental information and summaries used in this review were acquired from peer-reviewed articles in the relevant fields. The PubMed, Web of Science, Google Scholar, and China National Knowledge Infrastructure databases were searched for relevant articles. Information on the manual classification and selection of ginsenosides that protect against SCI is included in this review.

**Results:**

A literature survey yielded studies reporting several properties of ginsenosides, including anti-inflammation, anti-apoptosis, anti-oxidative stress, and inhibition of glial scar formation.

**Conclusion:**

In this review, we discuss the mechanisms of action of different ginsenosides that exert neuroprotective effects in SCI. These results suggest that after further verification in the future, ginsenosides may be used as adjunctive therapy to promote neurological recovery.

## Introduction

Spinal cord injury (SCI) is a devastating neurological disease that can lead to the loss of sensory and motor functions, quality of life, and social independence owing to the inability of the central nervous system (CNS) to adequately replace lost cells and connections (Sámano et al., 2021). The annual morbidity of SCI is approximately 15 to 40 cases per million globally and has increased with the development of modern society (Wang X. et al., 2021). According to the International Spinal Cord Society (ISCS), SCI can be divided into two main categories: traumatic and non-traumatic, with a prevalence of 90 and 10%, respectively (Gbd 2016 Neurology Collaborators, 2019). The former is usually caused by traffic accidents, sports injuries, and violent attacks, whereas nontraumatic SCI is mainly triggered by infection and vascular events (Badhiwala et al., 2020).

According to the different pathophysiological responses, the pathophysiological processes of SCI can be divided into primary and secondary phases. In the primary phase of SCI, the extent of injury, which causes cell necrosis, axonal transection, and local vascular loss around the injured area, is closely related to the strength of physical factors, such as compression, shearing, and acute stretch or tension (Anjum et al., 2020). The secondary injury phase reflects a multi-characteristic pathological process following the primary injury phase and lasts for several weeks. These include blood and vessel changes, oxidative stress, neuronal apoptosis, ionic deregulation, glutamate excitotoxicity, inflammation, fibroglial scarring, and cyst formation (Dimitrijevic et al., 2015). Neurologists and clinicians have long considered the secondary phase as a strategic therapeutic target to promote functional benefits.

Currently, various experimental therapeutic strategies for SCI are being researched in the fields of neurobiology, pharmacology, materials science, and other related scientific fields; however, they have not been implemented clinically (Albayar et al., 2019). Therefore, SCI treatment is limited to surgical decompression and high-dose intravenous methylprednisolone administration (Shinozaki et al., 2021). Surgery can efficiently decompress the damaged spinal cord, remove local irritants in due course, and stabilize the condition. However, recent clinical studies have shown that high doses of methylprednisolone may increase the incidence of many complications, including pneumonia, bedsores, and blood clots, which limit improvements in patients with SCI (Ter Wengel et al., 2019).

**FIGURE 1:**
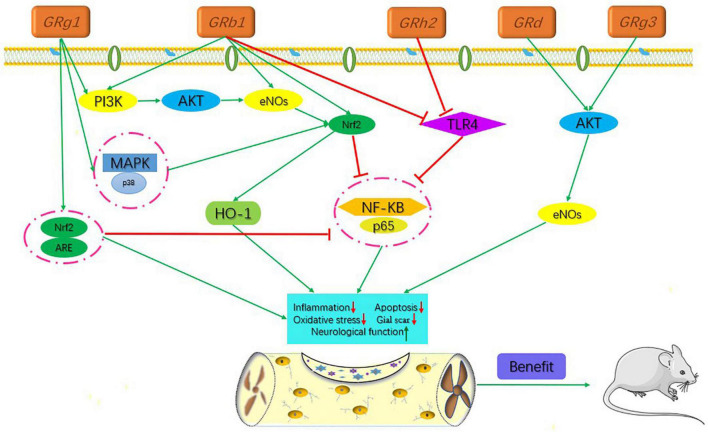
Signaling pathways of the different kinds of ginsenosides in the treatment of spinal cord injury.

Ginsenosides are found almost exclusively in the plant genus *Panax* but are mainly derived from *Panax ginseng* roots and processed via column purification or high-performance liquid chromatography (Lu et al., 2022). Approximately 40 ginsenoside compounds have been identified that have a wide spectrum of therapeutic effects on diabetes, cancer, stress, inflammation, immune stimulation, and cardiovascular diseases (Liu H. et al., 2020; Gong et al., 2022). Recent *in vivo* and *in vitro* studies have shown that all different ginsenosides subtypes had a significant impact on protecting against SCI. Therefore, with the vast array of ameliorative effects, such as antioxidant, neuroprotection, promotion of neurite outgrowth, and anti-inflammation, *P. ginseng* and its major components (Table 1 and Figure 1), ginsenosides, could potentially reduce secondary complications in patients with SCI. The present review discusses recent advances in the mechanisms of ginsenosides against SCI from different perspectives.

**TABLE 1:** Detailed information on the beneficial ginsenoside properties in the treatment of spinal cord injury.

Ginsenoside	Molecular structure	Anti-inflammatory	Anti-apoptotic	Anti-oxidant stress	Inhibition of glial scar formation	Signal path or receptor
GRb1	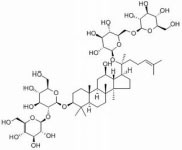	√		√		ERβ (Lee et al., 2021; Gong et al., 2022)
						
		√	√			TLR4/ NF-κB (Lü et al., 2019)
		√	√			GABA (Wang D. et al., 2021)
			√			AQP4 (Chen et al., 2020)
				√		eNOs/Nrf2/HO-1 (Li et al., 2019)
				√		PI3K/AKT (Luo et al., 2014; Tang et al., 2017; Liu X. et al., 2018)
				√		Nrf2/HO-1 (Ghafouri-Fard et al., 2022)
		√		√		PPARγ (Ye et al., 2016)
		√				Nrf2 and NF-κB (Li et al., 2017a)
GRg1	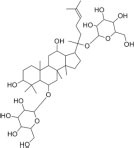		√		√	PI3K/AKT (Jang et al., 2016)
						
				√		Nrf2/ARE (Yi et al., 2019)
		√	√			MAPKp38/Nrf2/ NF-κB (Chu et al., 2019)
GRg3	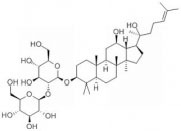		√			AKT/ eNOs (Fan et al., 2018)
						
GRh2	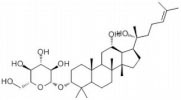	√		√		TLR4/NF-κB (Wang Y. et al., 2015)
						
GRd	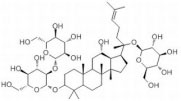		√	√		AKT/ERK (Xu X. et al., 2020)
						

## Specific varieties of ginsenoside ingredients

The active ingredients associated with the ginsenosides used against SCI are shown in Table 1 and Figure 1.

## Anti-inflammatory properties

**FIGURE 2:**
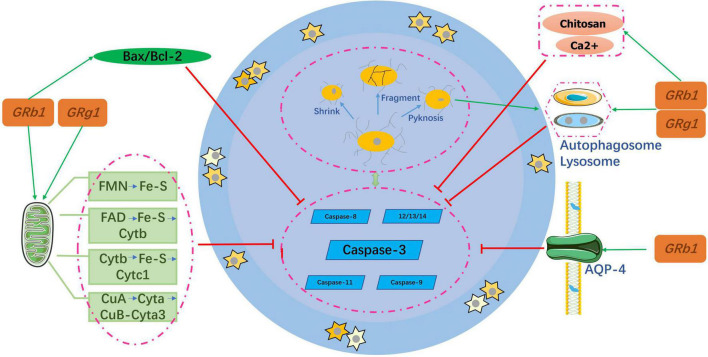
Mechanism of the anti-inflammatory properties of ginsenosides.

Inflammation is a crucial component of secondary injury after SCI and can be both beneficial and harmful to many cell types, including neutrophils, microglia, astrocytes, dendritic cells (DCS), blood-derived macrophages, and B and T lymphocytes (Zhang et al., 2017). When the spinal cord is injured, the damaged area forms an immune-induced microenvironment that recruits various cells that release numerous inflammatory cytokines. Inflammatory cells gather and infiltrate the spinal cord tissue, resulting in higher adhesion molecule expression and microvascular endothelial function. All of these factors trigger an inflammatory cascade reaction, which further aggravates spinal cord tissue damage (Mukhamedshina et al., 2017), as shown in Figure 2.

### Ginsenosides and microglia

Microglia are resident immune cells and macrophages. Upon injury, monocytes infiltrate spinal cord tissue and convert it into local macrophages. Macrophages and microglia have M1-like (pro-inflammatory) and M2-like (anti-inflammatory) phenotypes, respectively (Kroner et al., 2014). Currently, the most studied inflammatory factors are tumor necrosis factor-α (TNF-α), interleukin-1 beta (IL-1 β), and interleukin-6 (IL-6), which are indicative of the level of inflammation. Our ultimate goal was to facilitate the conversion from M1 to M2 phenotype.

Many studies have shown that different ginsenosides can significantly reduce the expression levels of the aforementioned inflammatory factors, in the microglia, through different key factors. Recent studies (Xu X. et al., 2020; Wang D. et al., 2021) found that both Rb1 and Rh2 can exert anti-inflammatory effects through the Toll-like receptor 4 (TLR4) and NF-κB signaling pathways in the microglia. Their results showed that the expression levels of IL-1β, IL-6, and TNF-α were significantly reduced. In contrast, Wang et al. found that another critical factor in Rb1 is microRNA-130b-5p. Another study (Jang et al., 2016) reported that nuclear factor erythroid-2 related factor 2 (Nrf2) could be the upstream signal factor for NF-κB pathways in the processing of Rb1, and Chen (Chen et al., 2020) showed that Rb1 reduces pro-inflammatory factors via the γ-aminobutyric acid (GABA) receptor. Similarly, other researchers concluded that the expression levels of the activation marker Iba-1 in the microglia were lower than before SCI, indicating that the ginsenosides acted as anti-inflammatory molecules (Kim et al., 2015, 2017).

In addition to the NF-κB signaling pathway in microglia, many researchers have focused on other pathways. A study (Su et al., 2016) demonstrated that notoginsenoside R1 (NG-R1) had a protective effect against oxLDL-induced endothelial cell injury by inhibiting the production of pro-inflammatory factors production via mitogen-activated protein kinases (MAPKs). In addition, the authors showed, for the first time, that anti-inflammatory effects were associated with the activation of peroxisome proliferator-activated receptor γ (PPARγ) protein expression and transcription levels.

Spinal cord injury treatment mainly involves the intravenous injection of hormones, after surgery, to effectively reduce spinal cord edema and cell damage; however, some inflammatory reactions inevitably occur. Shi et al. (2019) confirmed that Rg1 not only inhibits the pro-inflammatory cytokines TNF-α and IL-6, which are induced by hormones *in vivo* and *in vitro* but also increases the level of anti-inflammatory cytokine interleukin-4 (IL-4) in a variety of serum types.

### Ginsenosides and astrocytes

Astrocytes are not immune cells but play a pivotal role in the neuroinflammatory pathway. Upon injury, astrocytes transform into active astrocytes to enhance M1 chemokine production through the expression of TNF-α, IL-12, and IFN-γ. In addition to these inflammatory factors, if astrocytes are damaged, they lose the ability to regulate intracellular adhesion molecule (ICAM) and vascular cell adhesion molecule (VCAM) expression (Haroon et al., 2011).

After 21 days of simultaneous injection of Rg1 and Rb1 into rats, the intensity of glial fibrillary acidic protein (GFAP) and mRNA expression of GFAP was markedly inhibited (Lee et al., 2016). Furthermore, real-time PCR analysis demonstrated that the mRNA expression of ICAM-1 and VCAM-1 in the spinal cord were recovered.

### Ginsenosides and T lymphocytes

T lymphocytes play a vital role in adaptive immune responses, adopt different phenotypes, and contribute to the injury and repair processes. Teff cells control neuronal function by regulating the production of several pro-inflammatory cytokines and chemokines (Anjum et al., 2020). Treg cells, on the other hand, control the release of the anti-inflammatory cytokine, interleukin-10 (IL-10), and transforming growth factor-β (TGF-β). However, during SCI, Teff and Treg lose their balancing regulation, causing more Teff cell activities, resulting in a higher release of pro-inflammatory cytokines.

To the best of our knowledge, only Lee (Lee et al., 2016) has investigated the role of ginsenosides in T cell regulation. The results showed that Rg1 and Rb1 limited the recruitment and infiltration of Th1 and Th17 T cells into the spinal cord and inhibited the production of pro-inflammatory cytokines, such as IFN-γ and interleukin-17 (IL-17). Based on this study, ginsenosides may play an important role as an anti-inflammatory agent in SCI. However, more potential and innovative specific functional components need to be analyzed with a genetic database to facilitate the recovery of neurological function in the later stages, rather than limiting these usual cytokines.

## Anti-apoptotic properties

**FIGURE 3:**
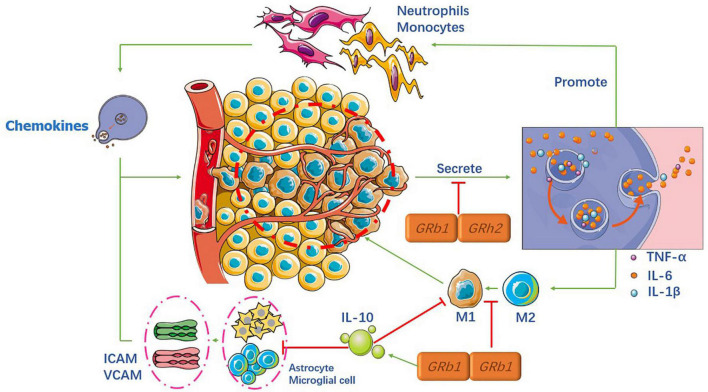
Mechanism of the anti-apoptotic properties of ginsenosides.

After SCI, the self-regeneration ability of neuronal cells is insufficient and the degree of self-repair is severely limited, which makes it difficult to recover nerve function at a later stage. Therefore, inhibition of neuronal cell apoptosis is an important part of the pathogenesis and repair process. Neuronal cell apoptosis usually refers to the programmed death of these cells and is the main cause of delayed spinal cord cell death after SCI. It is an active process of neuronal cell destruction, and its characteristics include cell shrinkage, genome fragmentation, chromatin aggregation, and nuclear pyknosis (Liu S. et al., 2018), as shown in Figure 3.

### Ginsenosides and the caspase family

The pathological process of neuronal apoptosis mainly involves a protease cascade mediated by members of the caspase family, including caspase-3, caspase-8 (Sobrido-Cameán and Barreiro-Iglesias, 2018), and caspase-9 (Keane et al., 2001). A few caspases, such as 11, 12, 13, and 14 have also been identified as specific apoptotic factors (Keane et al., 2001; Jin et al., 2011). Caspase-3 plays the most important role among these factors. The process of regulating neuronal apoptosis after the activation of Caspase-3 is regulated by B-cell lymphoma (Bcl). Zhao et al. (2018) confirmed that Rb1 reduced the Bax:Bcl-2 ratio, and caspase-3 and p-ASK-1 levels to protect nerve cells in the spinal cord of rats. Similarly, another study (Ahmed et al., 2016) found that Rb1 also inhibited caspase-3 and activated the anti-apoptotic gene BCL by phosphorylating the estrogen receptor. The conclusions of the two aforementioned studies are consistent with those obtained for Rg1 (Ke et al., 2014; Wang et al., 2014; Li et al., 2017b; Fan et al., 2018).

### Ginsenosides and autophagy

Autophagy is a major process mediating cell death (Sobrido-Cameán and Barreiro-Iglesias, 2018). As a recycling agent, it detoxifies unnecessary proteins and organelles by promoting lysosomal pathways. During SCI, abnormal activation of autophagosomes and lysosomes triggers rapid cell death (Yu and Fehlings, 2011). Luo et al. (2014) suggested that Rb1 might alleviate autophagic vacuoles and inhibit neuronal apoptosis induced by oxygen glucose deprivation (OGD) and transient ischemia. To further clarify the mechanism, the PI3K inhibitor LY294002 was used to prove that the anti-apoptotic effect was achieved through the PI3K/Akt signaling pathway. Similarly, Rg1 exerts neuroprotection via the same pathway as Rb1 (Yi et al., 2019).

### Ginsenosides and aquaporin-4

Aquaporin-4 (AQP4) is mainly distributed in the terminal feet of astrocytes and vascular endothelial cells and plays a key role in maintaining water balance. It is also an important protein involved in the development of spinal cord edema (Oklinski et al., 2016). When SCI occurs, the high expression of AQP-4 can lead to acute spinal cord edema, resulting in the inactivation and apoptosis of nerve cells, while the inhibition of AQP-4 expression can reduce cell water poisoning and the spinal cord edema index (Liu et al., 2015). Consequently, AQP-4 may be a key target in SCI management. Huang et al. (2015) demonstrated that the expression level of AQP-4 increased significantly after SCI and Rb1 could offset its growth. Compared with the spinal cord neurons of the untreated group, those in the treated group had neurons that were significantly complete and higher in number; such results were further corroborated by Li et al. (2019).

### Ginsenosides and mitochondria

Most of the energy required by the human body is provided by the mitochondria, and mitochondrial dysfunction causes neuronal death (Golpich et al., 2017). Xu M. et al. (2019) reported that GRb1 and GRg1 co-cultured with astrocytes significantly increased cell viability, decreased mitochondrial DNA (mtDNA) copy numbers, and weakened the mitochondrial membrane potential (MMP) depolarization. All these changes enhanced the mtDNA content. In addition, the two ginsenosides increased the activity of mitochondrial complexes I, II, III, and V and increased adenosine triphosphate (ATP) levels. Although GRb1 and GRg1 have different chemical structures, they are both involved in increasing the efficiency of mitochondrial oxidative phosphorylation in astrocytes.

In the last decade, researchers have found that if ginsenosides are wrapped with other ions, they may exert neuroprotective effects by inhibiting ion toxicity; this has become a new topical research area. In Guo et al. (2017) showed that using mechanical emulsification technology to prepare alginate chitosan microspheres by solidifying Rg1 combined with calcium ions and chitosan, can significantly improve the neuroprotective effect.

Therefore, in the future, ginsenosides should be combined with chemical materials to create a sustained release system that is placed in the injured area to increase local targeting and decrease toxic effects on other organs.

## Anti-oxidative stress

**FIGURE 4:**
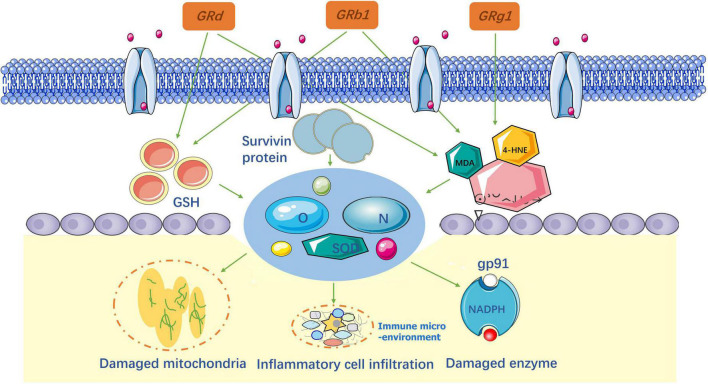
Anti-oxidative stress and lipid peroxidation mechanism of ginsenosides.

After SCI, oxidative stress destroys proteins, lipids, and DNA by producing reactive oxygen species (ROS) and reactive nitrogen species (RNS) in the spinal cord. ROS and RNS production increase ascorbic acid demand and alter the activity of antioxidant enzymes, such as superoxide dismutase (SODs), catalase, and glutathione (GSH). Therefore, neutralizing ROS and RNS is considered an effective way to reduce secondary injury in the treatment of SCI. The important biomarkers used to assess oxidative responses are 4-hydroxynonenal (4-HNE), malondialdehyde (MDA), and acrolein (Tsikas, 2017). When SCI occurs, the extracellular matrix is damaged and the products of membrane peroxidation are expressed. Consequently, these biomarkers can indirectly indicate damage caused by oxidative stress (Chio et al., 2021), as shown in Figure 4.

Researchers (Ye et al., 2016, 2019; Liu X. et al., 2018; Huo et al., 2019; Zhang et al., 2022) have confirmed that Rb1 and Rg1 significantly decrease the levels of ROS, RNS, and MDA after SCI to reduce oxidative stress in spinal cord tissues. In contrast, Ye and Huo found that the quantity of survivin protein and X-linked inhibitor of apoptosis protein (XIAP), around injured sites, increased in response to oxidative stress. In addition, serum catalase, superoxide dismutase (SOD), and GSH activity increased. At the same time, they validated that nuclear factor E2 (Nrf2) is the main cause of cellular defense against oxidative stress. This conclusion is in accordance with other researchers for Rg1 (Chu et al., 2019) and Rb1 (Ni et al., 2014). According to previous studies, ER-β was an important target for reducing oxidative stress, which was confirmed for Rb1 by Lü et al. (2019). In Cong and Chen (2016) first verified that Rd plays the same role as Rb1 and Rg1 in SCI treatment. The results of this study showed that Rd effectively reversed the redox imbalance in the spinal cord tissue by inhibiting the activation of the MAPK signaling pathway. Overall, the study evaluated a single ginsenoside in anti-oxidative stress (Sng et al., 2022). Given these results, it is necessary to consider using a combination of ginsenosides to improve secondary injury in patients with SCI.

## Inhibition of glial scar formation

**FIGURE 5:**
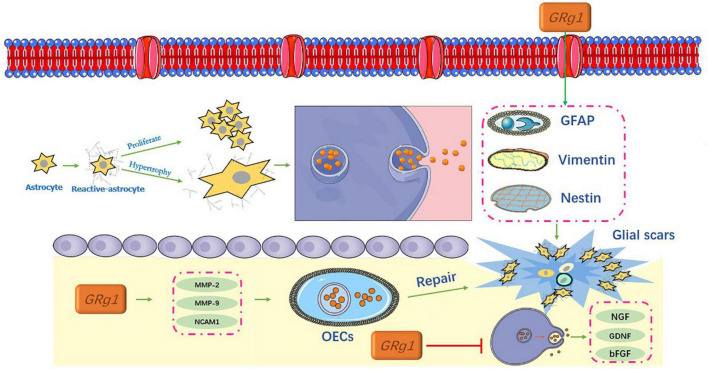
Inhibition of glial scar formation by ginsenosides.

Spinal cord injury triggers the formation of glial scar tissue around the injury epicenter, which is a multifactorial phenomenon that contributes to the damage of several neuronal populations of the injured spinal cord. After a scar is formed, it becomes a physical barrier in the physiological sense and secretes a variety of axon regeneration inhibitory factors (Robel and Sontheimer, 2016). Glial scars also form chemical barriers, as shown in Figure 5.

### Ginsenosides and astrocytes

After SCI, astrocytes become reactive. In the focal area, reactive astrocytes proliferate, hypertrophy, and migrate, and the cell processes are thick and overlap with each other. This is accompanied by an increased release of related proteins such as GFAP, vimentin, and nestin (Adams and Gallo, 2018). Together, these factors contribute to the formation of glial scars (Pekny and Pekna, 2014), and the process indicates a dynamic change (O’Shea et al., 2017).

Xu L. et al. (2020) conducted a study on glial fibrin degradation using Rg1. Their results showed that Rg1 significantly downregulated GFAP and chondroitin sulfate proteoglycans (CSPGs), and promoted the expression of nerve growth factor (NGF), glial cell line-derived neurotrophic factor (GDNF), basic fibroblast growth factor (bFGF), laminin (LN), and fibronectin (FN). Notably, these neurotrophic factors are involved in the regulation of neural differentiation and axonal regeneration in central peripheral neurons (Wada et al., 2018). In addition, they can promote the survival and repair of neurons to reduce the formation of neurocysticercosis disease and syringomyelia, and inhibit the formation of glial scars, which are beneficial for promoting spinal cord recovery (Lan et al., 2017).

### Ginsenosides and olfactory ensheathing cells

Several studies using SCI models of varying severity have shown that olfactory ensheathing cells (OECs) are glial cells capable of lifelong regeneration. The transplantation of OECs for the treatment of SCI has shown promising results (Zhou et al., 2019). Tang et al. (2017) reported that Rg1 upregulated the expression of migration factors that are related to OECs via the PI3K/Akt signaling pathway, including (MMP-2), matrix metalloproteinases-9 (MMP-9), and neural cell adhesion molecule 1 (NCAM1). Lu et al. (2010) obtained similar results for Rg1. These results indicate that Rg1 can not only promote the growth of OECs but also upregulate the expression of glial cell-derived neurotrophic factor and NGF, which may have great potential in OEC therapy. The migration and repair of OECs can further inhibit glial hyperplasia and scar formation, which is of great significance in SCI (Ingram et al., 2016).

However, until now, studies on ginsenosides that inhibit glial scar formation have been limited to a small number of neural cell types. Therefore, it is essential to determine the relationship between ginsenosides and more cell varieties, such as microglia, fibroblasts, and pericytes.

## Prospects of ginsenosides in protecting against spinal cord injury

To date, studies have shown the great potential of ginsenosides in the field of neuroprotective effects, and to some extent, revealed their related mechanisms. However, as a therapeutic strategy, there are still limited applications of ginsenosides. Thus, the following are our future prospects regarding research on ginsenosides. First, researchers have studied the mechanism of action of single ginsenosides in the treatment of SCI, and there are limited studies on combined ginsenosides application. In future studies, two or more ginsenosides could be used simultaneously to evaluate their neuroprotective effects. Second, the methods of administration were all by intraperitoneal injections, and absorption rates of other administration routes, such as gavage, were not investigated. More importantly, it is vital to know the concentration of the ginsenosides that reach the damaged spinal cord and whether it is associated with toxic side effects in other organs. Besides, extend the treatment to compare short and long-term efficacy after SCI. Third, there are only a few signaling pathways through which different ginsenosides act and the key proteins involved are not yet clear. In our opinion, we should increase the use of an applied gene database to identify the specific functional components of ginsenosides. Finally, ginsenosides should be combined with innovative chemical materials to improve their pharmacological role.

## Conclusion

Accumulating evidence has revealed the potential neuroprotective effects of ginsenosides on SCI, indicating that ginsenosides may be an adjuvant therapy in traditional Chinese medicines (TCMs). Based on this, this review concludes with the possible mechanisms of ginsenosides related to SCI and its limitations for future clinical translation. This review has focused on four possible mechanisms of SCI: anti-inflammatory, anti-apoptotic, anti-oxidative stress, and inhibition of glial scar formation. These mechanisms recommend ginsenosides as neuroprotective agents against many degenerative and traumatic diseases.

However, studies on the application of ginsenosides, in general, are still in their infancy. Many obstacles remain between basic research and clinical applications that need to be overcome in the future. To develop ginsenosides for use as a recognized clinical therapeutic approach, additional challenges need to be addressed, including a better-combined application of various ginsenosides, establishing the ideal method of administration and determining the absorption rate, comparing short and long-term efficacy, better understanding of the potential specific functional components, and more combinative forms with appropriate chemical materials to improve its efficacy.

## Author contributions

LQ reviewed the literature and contributed to the manuscript drafting and table and figures. JZ, JW, JA, and WX reviewed the draft. QL and YZ made critical revisions related to important intellectual content and analyzed and interpreted the imaging findings. All authors contributed to the article and approved the submitted version.
